# Photoperiod, but not testosterone, increases basal metabolic rate in house sparrows

**DOI:** 10.1093/iob/obag013

**Published:** 2026-04-02

**Authors:** H Galante, S J Lane, E K Elderbrock, J D Kittilson, K J Greenlee, T J Greives

**Affiliations:** Department of Biological Sciences, North Dakota State University, Fargo, ND 58108, USA; Department of Biological Sciences, North Dakota State University, Fargo, ND 58108, USA; Department of Biological Sciences, North Dakota State University, Fargo, ND 58108, USA; Department of Biological Sciences, North Dakota State University, Fargo, ND 58108, USA; Department of Biological Sciences, North Dakota State University, Fargo, ND 58108, USA; Department of Biological Sciences, North Dakota State University, Fargo, ND 58108, USA

## Abstract

Organisms in natural environments often undergo life history stage transitions that optimize behaviors (i.e., migration, reproduction, and foraging) with environmental conditions. These changes in behavior are facilitated by changes in physiology such as metabolism and energy production. Previous work on seasonally breeding songbirds observed that elevated levels of sex steroids during reproduction are accompanied by increases in basal metabolic rate (BMR), the minimum energy required to live for an adult organism. This increase in BMR is also associated with increasing daylengths in long-day seasonally breeding animals. Whether, and to what extent, the seasonal increase in BMR is a direct result of testosterone or in response to increases in duration and amount of activity occurring during longer days is not fully known. Experimental studies indicate that testosterone is capable of modulating mitochondrial function through activation of androgen and estrogen receptors within the mitochondria. However, whether testosterone directly influences BMR, and if it is related to changes in mitochondrial abundance remains unclear. Mitochondrial abundance can be quantified by assessing mitochondrial DNA copy number (mtDNAcn) which has been positively correlated with oxidative capacity and ATP production. Here, we assessed BMR of individual male house sparrows (*Passer domesticus*) during three experimental treatment periods: non-breeding short day photoperiods prior to hormonal manipulation (SD), followed by testosterone implants while still on non-breeding short days (SD + T), and then photostimulated on long days (LD) after implant removal, mimicking natural breeding conditions. We also collected blood samples to quantify testosterone and mtDNAcn of red blood cells (RBC mtDNAcn). Our results indicate testosterone did not directly alter BMR and that BMR was only elevated under longer daylengths associated with longer active periods. The total minutes of the day the birds were active increased under LD, thus indicating activity, and not increases in sex hormones, is likely responsible for the increases in BMR. We also observed no effect of treatment period on RBC mtDNAcn. Combined, the results from this study indicate that testosterone is not affecting BMR through changes in mitochondrial density (mtDNAcn) in red blood cells. However, changes in photoperiod affected BMR by either increasing daily activity or by stimulating the growth of reproductive tissues in seasonally breeding birds.

## Introduction

In seasonal environments, organisms transition between life history stages such as reproduction, growth, and migration, all of which involve changes in behavior and physiology. Changes in daylength, or photoperiod, coordinate these transitions (Dawson et al. 2001). These transitions are often costly, requiring changes in energy expenditure and metabolic rate. There are numerous studies that indicate seasonal phenotypic changes are related with seasonal fluctuations in metabolic rate associated with alterations in thermoregulatory demands (Mckechnie & Swanson 2010; Swanson 2010; Petit et al. 2013; McKechnie et al. 2015; Li et al. 2020). Additionally, previous work has indicated increases in metabolic rates occur during periods of reproduction (Weathers & Sullivan 1993; Piersma et al. 1995; Wells & Schaeffer 2012). Indeed, increased metabolic rate associated with reproduction has been positively associated with increases in reproductive hormone levels, like testosterone (Buchanan et al. 2001). These concurrent changes in metabolic rate across life history stages, along with seasonality, are well documented, but it is unclear which mechanisms (i.e., changes in thermoregulation, photoperiod, or sex steroids) are directly facilitating these changes.

To investigate variables associated with changes in metabolism and aerobic energy, researchers often measure an individual’s whole organismal basal metabolic rate (BMR), the minimum energy required for an adult organism to live at thermoneutrality in a resting, post-absorptive state (Hulbert & Else 2004; Swanson 2010). BMR is typically measured as the amount of oxygen consumed by an individual per unit of time via open flow through respirometry, thus representing an indirect measure of cellular aerobic performance and energy production (Hulbert and Else 2004; Lighton 2008). Aerobic cellular respiration, which provides an organism’s energy, is carried out in the mitochondria, and the density of mitochondria influences the amount of energy that can be produced, altering metabolic rates (Velando et al. 2019; Koch et al. 2021). Recently, Malkoc et al. (2021) and Casagrande et al. (2023) have indicated that mitochondrial oxidative capacity of blood is representative of whole organismal metabolic rate under non-stressful conditions. However, measuring metabolic rates of free-living animals can be time-consuming and stressful for the animals. A potential alternative approach that requires a simple blood sample that can be stored on ice and frozen until assayed could be quantifying tissue-specific mitochondrial abundance (i.e., blood), mitochondrial copy number (mtDNAcn), via real-time quantitative PCR (qPCR). While previous work has established that mtDNAcn from various tissues is indicative of cellular oxidative capacity and ATP production along with being related to development and aging (Gross et al. 1969; Williams et al. 1986; Short et al. 2005; Rocher et al. 2008; Pyle et al. 2015; Sun et al. 2016; Salminen et al. 2017; Velando et al. 2019), it remains to be investigated how blood mtDNAcn is related with whole organismal metabolic rate. Additionally, mitochondrial densities in the blood (RBC mtDNAcn) and cellular respiration rates are plastic traits and have previously been observed to change together between reproductive stages, allowing for the fine tuning of energy expenditure to appropriately match energy demands (Stier et al. 2019). The connection between mitochondrial respiration and mitochondrial abundance highlights the importance of mitochondria in shaping life history stages on an organismal level, such as reproduction.

Reproduction is a life history stage associated with increased metabolic demands (Green et al. 2013). Many vertebrates, such as temperate zone songbirds, experience extreme fluctuations in temperature and daylengths along with displaying distinct seasonal breeding patterns (Dawson et al. 2001; Leska & Dusza 2007). Specifically, increases in photoperiod stimulates reproductive maturation and annual peaks in reproductive hormones (estrogen and androgens) (Wingfield & Farner 1976; Nottebohm et al. 1987; Wingfield et al. 1990; Foerster et al. 2002; Valdez et al. 2014). These seasonal transitions are associated with increases in metabolism, especially in males. For instance, when exposed to long breeding daylengths, male white-footed mice (*Peromyscus leucopus*) exhibited a positive correlation between BMR and estimated testis volume (Kaseloo et al. 2012). Additionally, in male house sparrows, increases in testosterone and increased badge size were associated with elevated BMR, indicating a cost for male sexual signaling (Buchanan et al. 2001). Independent of maturation of gonads, cost of sexual signaling, and increases in reproductive steroid hormones, the longer daylengths experienced during spring and summer also allows for longer active days, which can be positively correlated with daily energy expenditure and BMR (Tinbergen & Dietz 1994; Wikelski et al. 1999; Dawson et al. 2001; Poesel et al. 2006; Adkins-Regan 2007; Kaseloo et al. 2012; Sadowska et al. 2013; McDonnell et al. 2019). Combined, these physiological and behavioral changes, related to seasonal fluctuations in daylength, leads to increases in energy demand during long days (Weathers & Sullivan 1993; Anava et al. 2002; Swanson 2010). However, few studies disentangle whether photoperiodic changes in duration of activity/behavior or seasonal photostimulated increases in reproductive hormones are responsible for changes in BMR during reproduction, irrespective of seasonal thermoregulatory fluctuations.

Previous research has indicated that sex hormones and photoperiod-driven changes in reproductive state (i.e., presence or absence of gonads, a energetically expensive tissue) are associated with changes in BMR in males (Hanssler & Prinzinger 1979; Chappell et al. 1999; Buchanan et al. 2001; Anderson 2006; Trivedi et al. 2006; Kaseloo et al. 2012). For example, castration significantly decreased BMR in Japanese quail (*Coturnix japonica*), (Hanssler and Prinzinger 1979), while androgen implantation increased BMR in Mozambique tilapia (*Oreochromis mossambicus*) (Ros et al. 2004), which both occurred under long breeding photoperiods. Additionally, similar patterns between BMR and reproductive hormones have been exhibited in species that experience dissociated gonad development and reproductive behavior like the red-sided garter snake (*Thamnophis sirtalis parietalis*). Male red-sided garter snakes experience gonadal maturation and spermatogenesis that occurs outside the breeding season under short days, when testosterone levels are high, which coincides with seasonal elevations in metabolic rates (Crews et al. 1987; Krohmer & Grassman 1987). Conversely, both castrated and intact male white crowned sparrows on long days (*Zonotrichia leucophrys*) receiving testosterone implants displayed a reduction in resting metabolic rate. The authors suggested that this reduction in resting metabolic rate in response to testosterone implants functioned to offset the increased metabolic cost of increased activity associated with the longer active daylight periods (Wikelski et al. 1999). This study however did not investigate the effects of hormonal manipulation under short photoperiods to test how testosterone is directionally affecting (i.e., increasing or decreasing) metabolic rate under different photoperiods (Wikelski et al. 1999).

Whether daylength associated changes in BMR are facilitated via changes in mitochondrial abundance remains understudied. If sex steroid hormones are acting to increase BMR, one potential way could be via influencing mitochondrial biogenesis and abundance. Sex steroids help maintain mitochondrial function, abundance, and biogenesis through the presence of estrogen receptors and androgen receptors on the outer membrane of the mitochondria, as well with hormone response elements within the mitochondrial and nuclear DNA, where hormones bond to their respective receptor to bind directly to DNA (Tcherepanova et al. 2000; Chen et al. 2004; Mattingly et al. 2008; Ivanova et al. 2011; Velarde 2014; Ahmad & Newell-Fugate 2022). In fact, estrogen receptors have been found on the surface of red blood cells (Vona et al. 2019). Further, hematocrit levels are positively correlated with testosterone levels, and blocking androgen receptors with flutamide decreases hematocrit (Kobayashi et al. 2022). Together, these suggest that sex steroids are capable of influencing mtDNAcn in red blood cells.

**Fig. 1:**
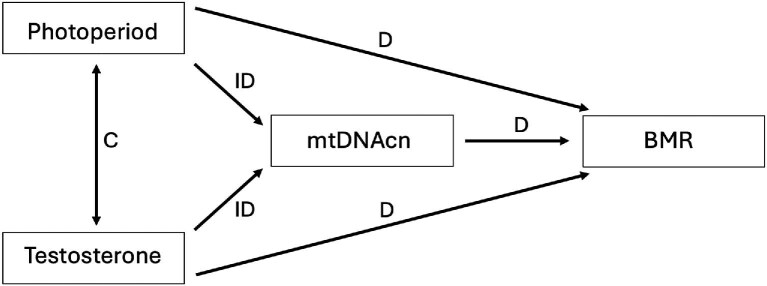
Predicted effects of photoperiod and testosterone on RBC mtDNAcn and BMR. Changes in testosterone and photoperiods are predicted to have either a direct effect (D) or an indirect effect (ID), mediated by changes in RBC mtDNAcn, on BMR (i.e., elevated circulating levels of androgens or longer photoperiods increase RBC mtDNAcn which in turn increases BMR). Changes in RBC mtDNAcn are predicted to directly (D) effect BMR. Photoperiod and testosterone are correlated (C) since increases in testosterone co-occur with longer photoperiods under natural conditions in house sparrows.

While previous observations indicate that photoperiod-induced changes in reproductive status are likely factors affecting BMR, it is not clear if the changes in BMR are directly altered in response to longer active daylengths (increased activity), or if they are changing in response to changes in sex hormones, irrespective of thermoregulatory needs. Here we test the hypothesis that reproduction-associated increases in BMR in seasonally breeding birds are mediated by increases in sex hormones, specifically testosterone. We hypothesize that these testosterone-induced increases in BMR are indirectly mediated via testosterone by increasing blood mitochondrial density (RBC mtDNAcn). To test our hypotheses, we measured the BMR of captive male house sparrows (*Passer domesticus*) under non-breeding short photoperiods before and after testosterone implantation, and again after stimulation with long photoperiods following testosterone implant removal, mimicking natural breeding daylengths; all while held under thermoneutral conditions. We predicted that, regardless of daylength, birds with higher levels of testosterone, both from exogenous (implants) during short days and endogenous (longer photoperiods) sources, would have higher BMR and elevated levels of blood mtDNAcn compared to BMR and blood mtDNAcn sampled during unmanipulated short days (Fig. 1). Additionally, our study investigates if blood mtDNAcn is representative of whole organismal BMR, potentially offering a less invasive sampling technique for measuring metabolic traits.

## Material and methods

### Experimental animals

Sixteen adult male house sparrows were captured with mist-nets from free-living populations residing in Fargo, ND, USA in late November 2022. Birds were housed individually in cages (59.7 × 39.4 × 30.5 cm) in two temperature and light-controlled rooms. All birds received food (millet and deshelled sunflower mix, grit, mealworms, a dog food mixture with hard-boiled eggs and carrots) and water *ad libitum*. Birds’ weights were measured weekly to assess health. Two birds developed an illness and were removed from the study at the time point that symptoms were exhibited, thus the final sample size was 14.

### Assessment of cloacal protuberances & testes mass

The width (mm) of the bird’s cloacal protuberance (CP) was measured to the nearest tenth using calipers (SPI plastic dial caliper 150 mm, metric dial of 0.1 mm) prior to each BMR measurement (Boersma & Davies 1987). Cloacal tissue is androgen sensitive and grows in response to the presence of androgens. We used CP size to verify the effectiveness of our experimental treatment and to determine the length of the post-implant period (see experimental design) (Balthazart et al. 1980; Massa et al. 1980). At the end of photostimulation, birds were euthanized with an overdose of isoflurane. Testes were removed and weighed on an electric scale to the nearest 0.0001 g to confirm that birds were photostimulated (Lombardo & Thorpe 2009). Testes mass of these birds following photostimulation was 0.3458 ± 0.1478 g with a range of 0.1408–0.6709 g (Galante et al. 2024).

### Experimental design

Once brought into captivity, all birds were fitted with a BitTag accelerometer to record daily activity (Brown et al. 2023). Accelerometers were attached via a leg loop harness made from 1 mm elastic cord (Naef-Daenzer 2007). Birds were initially held on short-day light cycles (SD-8L:16D, 08:00–16:00) for 2 weeks prior to any manipulations to allow time for adjustment to captivity (Buttemer & Astheimer 2000). Housing rooms were kept between 21 and 22°C for the duration of the experiment. After the 2-week adjustment period, we took baseline BMR measurements (see below BMR set up) (Buttemer & Astheimer 2000). The following night, we collected a baseline blood sample at 21:00 for RBC mtDNAcn and testosterone levels (see blood sampling below). The day following baseline blood sampling, all birds received a single 11 mm silastic subcutaneous testosterone implant packed with crystalline testosterone (Sigma) (1.47 mm in diameter; 1.96 mm outer diameter; Dow Corning), sealed at both ends with Sil-Bond 100% silicone sealant (Silco Inc.; RTV 4500) on their flank (Hau et al. 2004). Photoperiod was maintained at 8L:16D for 3 weeks following implantation. After those 3 weeks a second BMR was performed. A second blood sample was taken at 21:00 the next night to measure testosterone to confirm effectiveness of the implants and to assess RBC mtDNAcn.

Implants were removed the following day after the second blood sample was collected. Birds were allowed to clear the exogenous testosterone for 3 weeks following removal of implants while remaining on 8L:16D. To confirm that testosterone had been cleared after this 3-week period, we collected a blood sample (between 11:00 and 13:00) and confirmed that CP widths were reduced and did not differ from their baseline CP widths at the start of the study.

**Fig. 2:**
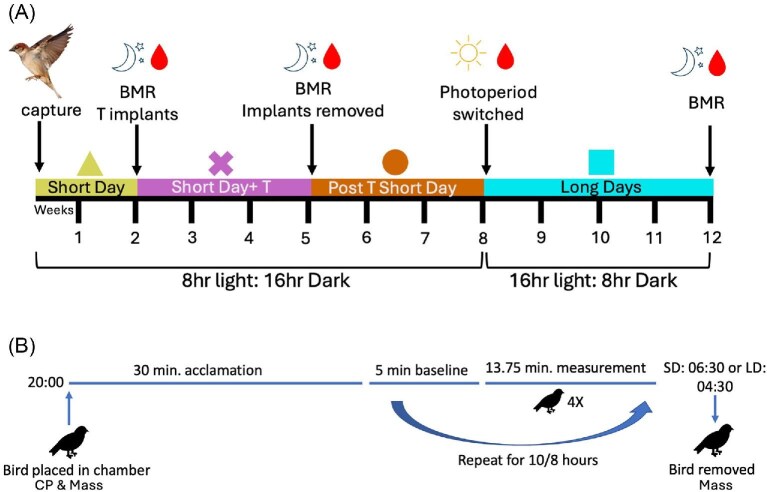
(A) Experimental design, each color represents each treatment period (yellow/triangle: short days [SD]; pink/X-mark: short days with testosterone implants [SD + T]; dark orange/circle: implants removed still on short days [Post T SD]; light blue/square: long days [LD]). Blood sampling is indicated at what time of day it was collected at the end of each treatment period and were taken 24 h after nighttime BMR was conducted. Additionally, short days consisted of 8:16 light/dark cycles and long days consisted of 16:8 light/dark cycles. (B) Nighttime BMR respiratory measurement scheme.

Following confirmation that CP size had been reduced, birds were photostimulated (LD-16L:8D, 04:00–20:00) for 4 weeks. This period of photostimulation has been demonstrated to elevate endogenous production of testosterone (Farner et al. 1966; Small et al. 2007; Needham et al. 2016). At the end of the 4-weeks a third BMR was measured along with a blood sample taken at 22:00 the following night to confirm that photostimulation had increased circulating testosterone levels and to measure RBC mtDNAcn (Fig. 2A).

### Metabolic chambers

Metabolic chambers were composed of airtight 1.89-L clear glass jars with tubing attached to the multiplexer and were fitted with a perch so birds could rest comfortably (Buttemer et al. 2022). Perches were positioned 10 cm above the bottom of the chamber (15 cm from the top). The bottoms of the chambers were covered with hardware cloth (4 mm × 4 mm square grid) placed 5 cm below the perch and 5 cm above the bottom of the jar. Below the mesh, 2 cm of medical grade mineral oil was added to absorb fecal matter to minimize any increase in water vapor to the air, which would influence measures of excurrent oxygen (Jacobs & McKechnie 2014; Boyce et al. 2020).

### Basal metabolic rate measurements

Metabolic measurements were performed during the sparrow’s fasted nocturnal rest phase at room temperature ∼22°C, which is at the lower end of the thermoneutral zone (20–35°C) for northern populations of house sparrows, in an insulated chamber (Percival Scientific)( Hudson & Kimzey 1966; Kendeigh 1969). However, due to reported population differences in thermoneutral zones and critical lower temperatures, our test of metabolic rate measured at 22°C could also be considered resting metabolic rate (Hart 1962; Nzama et al. 2010). To achieve a post-absorptive fasted state, food was removed 2 h before lights off (at 14:00 during short-day light cycles and at 18:00 during long-day light cycles). In both short- and long-day conditions, sparrows were transferred to metabolic chambers at 20:00 and allowed to acclimate for 30 min prior to starting the measurements (Arens & Cooper 2005). Each bird was weighed immediately before placement into the chamber and then had their BMR measured once per hour for a 13.75-min interval along with a 5-min baseline, O_2_ sampling rate consisted of 5-s intervals for both baseline and BMR measurements (Martin et al. 2003). Measurements occurred during the resting phase for each photoperiod treatment and were repeated each hour for 10 h during short days and each hour for 8 h under long days. After BMR measurements were completed, birds were weighed and placed immediately back into their cages (SD-06:30 and LD-04:30) (Fig. 2B).

CO_2_-free air (Balston purge gas generator, Parker, Cleveland, OH, USA Purged gas) was scrubbed of H_2_O with Drierite (Acros Organics) and Ascarite II (Thomas Scientific) upstream from the respirometry chambers and was pushed through the chambers at a flow rate of 600 mL/min using a mass flow controller (MFC-4, Sable Systems, Las Vegas, NV, USA). CO_2_ and H_2_O from downstream excurrent air from the chambers was removed with Ascarite II and magnesium perchlorate (Fisher) prior to the oxygen analyzer (Oxzilla FC-2 Differential Oxygen Analyzer, Sable Systems, Las Vegas, NV) (Buttemer et al. 2008; Malkoc et al. 2021). An 8-channel multiplexer (Sable Systems, Las Vegas, NV) was used to direct airflow to the oxygen analyzer for individual metabolic chamber measurements, as well as directing airflow in and out of the metabolic chambers when not being measured. This set up allowed us to sample a maximum of seven birds, on average four birds were measured per night, with one empty control chamber. Sable Systems hardware (UI2) and software (Expedata) were used to digitize and record % oxygen in the airstream.

### Basal metabolic rate calculations

BMR was calculated from the two lowest 13.75-min periods of % oxygen consumption from the last half of the respirometry run (SD: 01:30–06:30, LD: 00:30–04:30) (Buttemer et al. 2022). Percent oxygen consumed was calculated by subtracting the mean % of oxygen from the baseline reading, then divided by 100 and multiplied by the flow rate (600 mL/min) to get O_2_ mL/min consumed, which was converted to O_2_ mL/h consumed (Lighton 2008). Finally, O_2_ mL/h was divided by the average mass of the bird from the start and end of the run to determine O_2_ mL/h/g.

### Blood sampling

Blood (∼115 µL) was collected with heparinized microhematocrit capillary tubes (Fisher Scientific) from the brachial vein following venipuncture with a 26-g needle and immediately placed on ice. Plasma and red blood cells were separated by centrifuging the samples at 10,600×g for 10 min at 4°C. Plasma was extracted and stored at −20°C until assayed for testosterone and red blood cells were stored at −80°C until DNA extraction for RBC mtDNAcn.

Sampling at 21:00 (SD and SD + T) and 22:00 (LD) was conducted using red-illuminated headlamps. Samples were collected during the dark phase of the bird’s circadian rhythms during a similar timeframe as when BMR was being assessed and when testosterone levels exhibit a daily peak in this (and other songbird) species (Needham et al. 2016; Greives et al. 2021). Birds were captured from their home cages and removed to a separate room for sampling. One bird was sampled at a time, with birds remaining in their home cage until sampling. Birds were kept under red light, and all nighttime samples were collected within an average of 7.91 (range: 3–22) min upon first entrance to the room. Following sampling, birds were returned to their cages. Blood sampling to confirm testosterone clearance following implant removal was conducted under normal lighting conditions between 11:00 and 13:00. Testosterone levels for this current study were assessed and reported in Galante et al. (2024) which found that treatment period had a significant effect on circulating levels of testosterone. Both testosterone treatment (SD + T) and long-day photoperiods (LD) increased the average testosterone level significantly, 13.22 ± 1.84 ng mL^−1^ and 2.22 ± 0.47 ng mL^−1^ respectively, compared to short days prior to any manipulations (SD) that had an average of 0.11 ± 0.01 ng mL^−1^. Post-implant removal prior to phototstimulation (Post T SD), birds had an average of 0.36 ± 0.06 ng mL^−1^ circulating testosterone (Supplementary Table S1)(Galante et al. 2024).

### Mitochondrial copy number

Real time qPCR was used to measure mtDNAcn (mtDNA/S) from red blood cells by quantifying the amount of mitochondrial DNA relative to a single copy reference nuclear gene (Furda et al. 2012). Mitochondrial DNA and nuclear DNA were extracted from ∼3–4 µL of packed red blood cells using a NucleoSpin blood kit according to manufacturer’s instructions (Macherey-Nagel). Glyceraldehyde-3-phosphate dehydrogenase (GAPDH) was used as the single nuclear reference region previously validated for house sparrows (Velando et al. 2019; Heidinger et al. 2021) and the mitochondrial gene cytochrome B (CytoB) was used to amplify mtDNA (Darr et al. 2017). Primer sequences for CytoB were designed in IDT primer design from the complete annotated house sparrow mitochondrion found on NCBI (KM078784.1) (Supplementary Table S2).

For real time qPCR, reactions were performed in triplicate at a total volume of 15 µL, with 10 ng of template DNA, primers at a final concentration of 100 nM and 7.5 µL of Sybr Green Super Mix (Quanta Bio) (Velando et al. 2019). The mitochondrial gene, CytoB, and the reference gene, GAPDH, were performed on different plates. Each plate contained a standard curve with 250, 50, 10, 2, and 0.4 ng of template DNA, along with a no template control and a 10 ng reference standard. The real time amplification conditions for GAPDH were: 10 min at 95°C, followed by 40 cycles of 30 s at 95°C, 30 s at 60°C, then finalized with 1 cycle of 1 min at 95°C, 30 s at 55°C, and 30 s at 95°C. For CytoB the qPCR conditions were as follows: 10 min at 95°C, followed by 40 cycles of 15 s at 95°C, 30 s at 57°C, 30 s at 72°C, then finalized with 1 cycle at 1 min at 95°C, 30 s at 55°C and 30 s at 95°C. Real time qPCR reactions were performed in a Mx3000P (Agilent Technologies). Melt curve analyses confirmed that only a single amplicon was created for GAPDH and CytoB from the qPCR reactions. The average reaction efficiency for each gene was 99.3% for GAPDH and 102.9% for CytoB. For calculating the RBC mtDNAcn, the average of triplicate values were used to calculate mtDNA/S ratio for each sample relative to the reference gene according to the following formula: 2^(-ΔΔCt)^, where ΔΔCt= (Ct^CytoB^−Ct^GADPH^) focal sample − (Ct^CytoB^−Ct^GADPH^) reference standard (Bize et al. 2009; Heidinger et al. 2012).

### Behavioral data and total activity calculations

BitTags are programmable accelerometers that continually measure and record behavioral activity. Percent activity for each minute was calculated by tracking acceleration changes (Brown et al. 2023). BitTags were programmed using the same settings as in Galante et al. (2024). Total activity was calculated by converting the % activity per minute to total minutes active per day by first summing the % activity per minute for each day to get the total activity % per day. The total activity % per day was then divided by 144,000, which represents that maximum possible % activity per day, to get the proportion of the day birds were active. Next, the proportion of the day birds were active was multiplied by 1440, total number of minutes in a day, to get total minutes active per day.

### Statistical analyses

All analyses were performed in R studio (R Core Team 2022, Version 4.2.2).

### Effect of treatment on basal metabolic rate, cloacal protuberance size, total daily activity, and mitochondrial DNA copy number

Linear mixed effects models were used to investigate the effect of treatment on mass corrected BMR, non-mass corrected BMR, cloacal protuberance (CP), total daily activity and RBC mtDNAcn, as response variables, using the package “lme4” (Bates 2010; Bates et al. 2015). Model assumptions were assessed using the “check_model” function from the performance package (Lüdecke et al. 2021). For models that assessed mass corrected BMR, CP, total daily activity and RBC mtDNAcn, we then tested the significance of fixed effects of linear mixed models using a Type III ANOVA, and the statistical significance level of all tests was set at *α* = 0.05. Degrees of freedom were calculated for all models using the Satterthwaite’s method. Specifically for the model investigating non-mass corrected BMR, we used a Type II Wald chi-square to test the significance of fixed effects of linear mixed models with a statistical significance level set at *α* = 0.05 test due to a significant Levene’s test. Following significant main effects identified by the linear mixed models, we performed a post hoc pairwise comparison using the “emmeans” package with a Holm-Bonferroni adjustment for all comparisons (Lenth 2016, 2023).

For mass corrected BMR and total daily activity, treatment period (short days prior manipulation [SD], short days with testosterone implants [SD + T], and long days [LD]) was a categorical variable included as a fixed effect and individual identity was set as a random effect. The model for CP, treatment period was used as a categorical fixed effect with the addition of short days post-implantation (Post T SD) to the above sampling points from the mass corrected BMR and total daily activity models, individual identity was set as a random effect as well. For red blood cell mitochondrial DNA copy number (RBC mtDNAcn), both treatment, a categorical variable, and qPCR plate number (samples were run on three plates), a numerical discrete variable, were included as fixed effects, while individual identity was set as a random effect. Following a significant main effect for BMR, CP size, and total daily activity, a pairwise post-hoc comparison between treatment periods was conducted. Additionally, for non-mass corrected BMR, both treatment, a categorical variable, and mass, a numeric continuous variable, were included as fixed effects while individual identity was set as a random effect.

### Relationship between basal metabolic rate and mitochondrial DNA copy number

To test the relationship between mass corrected BMR, non-massed corrected BMR and RBC mtDNAcn we fitted a linear mixed-effects model with an interaction between fixed effects. Fixed effects included treatment period (short days prior manipulation [SD], short days with testosterone implants [SD + T], and long days [LD]), as a categorical variable, and RBC mtDNAcn as a continuous numerical variable, along with individual identity set as a random effect for both massed corrected and non-mass corrected BMR. However, non-mass corrected BMR additionally included mass, a numeric continuous variable, as a fixed effect. Following a significant interaction, a pairwise post-hoc test was performed using “emtrends” from the “emmeans” package to estimate slopes predicted by the model (Lenth 2016, 2023). We then did a pair-wise comparison between groups using the “pairs” function. Treatment period was grouped by factor, RBC mtDNAcn was denoted as the continuous independent variable and BMR was the response variable.

### Repeatability of basal metabolic rate and mitochondrial DNA copy number

Repeatability was tested using the function rpt from the “rptR” package with the number of bootstraps set to 1000 and datatype set to gaussian (Stoffel et al. 2017). To test if mass corrected BMR, non-mass corrected BMR and RBC mtDNAcn were repeatable across all treatments, identity was included as a random effect. Fixed effects for RBC mtDNAcn included treatment period (short days prior manipulation [SD], short days with testosterone implants [SD + T], and long days [LD]) as a categorical variable and qPCR plate number (samples were run on three plates) as a numerical discrete variable. Fixed effects for mass corrected BMR only included treatment period (short days prior manipulation [SD], short days with testosterone implants [SD + T], and long days [LD]) as a categorical variable. For non-mass corrected BMR treatment (short days prior manipulation [SD], short days with testosterone implants [SD + T], and long days [LD]), a categorical variable, and mass, a numeric continuous variable, were included as fixed effects. Additionally, repeatability for mass corrected BMR and non-mass corrected BMR was assessed between short days and short days with testosterone implants, to test repeatability within the same photoperiod, as well as between short days with testosterone implants and long days, to test repeatability with birds experiencing increased levels of testosterone from implants or photostimulation.

## Results

### Treatment effect on cloacal protuberances

**Fig. 3:**
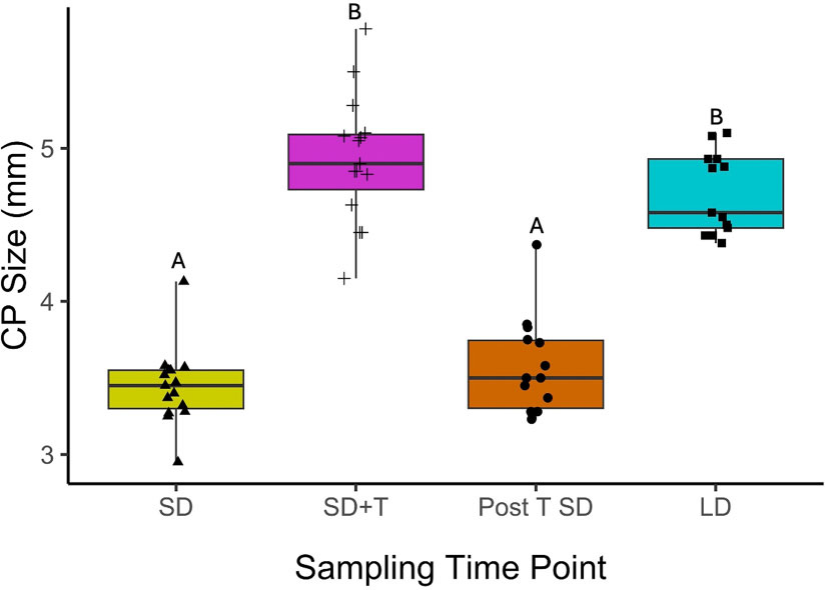
Photoperiod and testosterone implants had an effect on individual CP size (mm) across treatments [Short days [SD]; Short days with testosterone implants (SD + T); Short days post implant removal [Post T SD]; and Long days [LD]). Letters indicate significant differences between groups. Data points represent a single individual for each sampling time point.

Treatment had a significant main effect on CP size (F_3, 40.07_ = 114.82, *P* < 0.0001; Fig. 3). Pair-wise post-hoc comparisons found that CP size after implantation (SD + T) and photostimulation (LD) were significantly larger compared to short days prior to any manipulation (SD) and after implant removal (Post T SD), (post-hoc: SD-(SD + T) t(_df_  _= 39.08_)= −15.02, *P* < 0.0001; SD-LD t(_df= 40.02_)= −12.23, *P* < 0.0001; (SD + T)-post T SD t(_df= 39.64_) = 13.40, *P* < 0.0001; post T SD-LD t(_df= 39.48_)= −10.85, *P* < 0.0001, Supplementary Table S3). Prior to manipulation, short days exposed sparrows (SD) had an average CP size of 3.44 ± 0.07 mm. Testosterone treatment during short-day photoperiod (SD + T) increased the average CP size to 4.93 ± 0.11 mm. Following removal of testosterone implant while on short days (Post T SD), average CP size decreased to 3.57 ± 0.08 mm. After birds were moved to long day photoperiods (LD), the average CP size increased to 4.7 ± 0.07 mm. Weekly CP sizes across all treatment periods are additionally reported in Galante et al. (2024).

### Treatment effect on basal metabolic rate

**Fig. 4:**
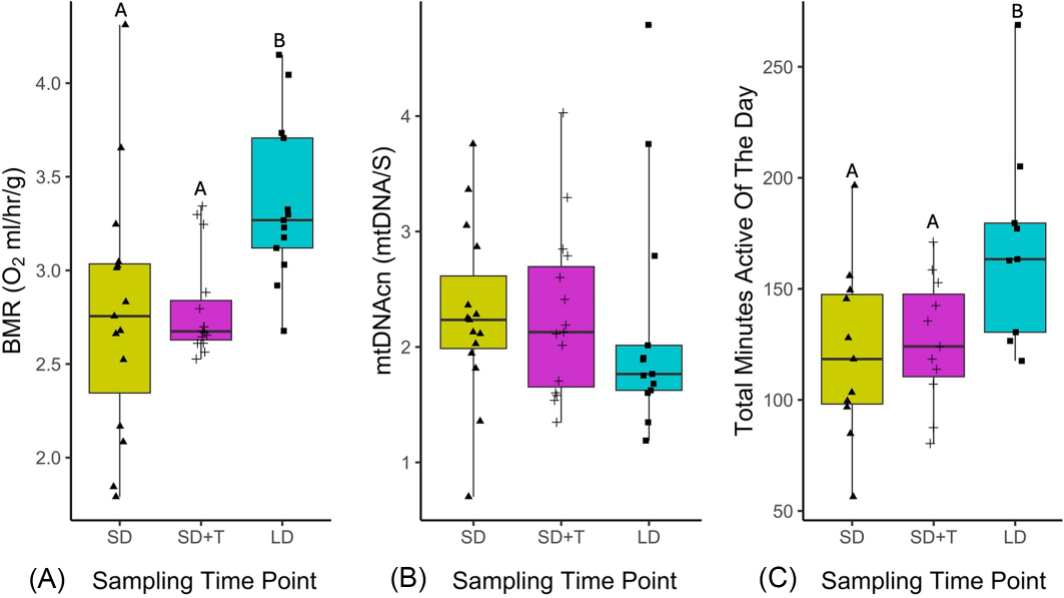
(A) Individual mean mass corrected BMR across treatments (Short days [SD]; Short days with testosterone implants [SD + T]; Long days [LD]). Data points represent the average of the two lowest BMR measurements from the last half of the BMR measuring time frame for each individual for each treatment period. Letters indicate significant differences between groups. (B) Individual RBC mtDNAcn across treatments (Short days [SD]; Short days with testosterone implants (SD + T); Long days [LD]). Data points represent a single individual for each sampling time point. (C) Daily individual mean of total daily activity across treatments (Short days [SD]; Short days with testosterone implants (SD + T); Long days [LD]). Data points represent the average total minutes active per individual for each treatment period. Letters indicate significant differences between groups.

There was a significant main effect of treatment period on mass corrected BMR (F_2, 26.55_ = 6.95, *P* = 0.0037; Fig. 4A). Pair-wise post-hoc comparison revealed that mass corrected BMR was elevated on long days (LD) compared to all other treatments (Post-Hoc: SD-LD t(_df= 27.19_)= −3.31, *P* = 0.0079; (SD + T)-LD t(_df= 27.19_)= −3.20, *P* = 0.0079; Supplementary Table S4). No other significant pair-wise differences were identified. Individual mean of massed corrected BMR during short days prior to manipulation (SD) was 2.78 ± 0.17 O_2_ mL/h/g and was 2.79 ± 0.07 O_2_ mL/h/g during testosterone treatment on short days (SD + T). During photostimulation (LD), individual mean of massed corrected BMR increased to 3.36 ± 0.12 O_2_ mL/h/g. For non-mass corrected BMR, there was a significant main effect of treatment period as well (*X*^2^ [2, N = 43] = 14.31, p = 0.0008). Pair-wise post-hoc comparison revealed that non-mass corrected BMR was elevated on long days (LD) compared to all other treatments (Post-Hoc: SD-LD t(_df= 27.02_)= −3.35, *P* = 0.0072; (SD + T)-LD t(_df= 26.84_)= −3.26, *P* = 0.0072; Supplementary Table S5). No other significant pair-wise differences were identified. Individual mean of non-mass corrected BMR during short days prior to manipulation (SD) was 76.06 ± 4.57 O_2_ mL/h and was 76.53 ± 1.37 O_2_ mL/h during testosterone treatment on short days (SD + T). During photostimulation (LD), individual mean of non-massed corrected BMR increased to 92.02 ± 3.34 O_2_ mL/h (Supplementary Fig. S1).

### Treatment effect on mitochondrial DNA copy number

There was no signficant effect of treatment period on RBC mtDNAcn (F_2, 24.86_ = 0.81, *P* = 0.4543; Fig. 4B). The mean relative abundance of RBC mtDNAcn was 2.28 ± 0.20 mtDNA/S for short days prior to any manipulation (SD) and 2.28 ± 0.19 mtDNA/S for testosterone implantation (SD + T). On long days (LD), mean relative abundance of RBC mtDNAcn was 2.16 ± 0.29 mtDNA/S.

### Treatment effect on total daily activity

Treatment had a significant effect on total active minutes per day (F_2,581.75_ = 72.1, *P* < 0.0001; Fig. 4C). Pair-wise post-hoc comparisons indicated the total daily activity was elevated on long days (LD) compared to all other treatments (Post-Hoc: SD-LD t(_df_  _= 583.32_)= −10.91, *P* < 0.0001; (SD + T)-LD t(_df=583.71_) = −10.32, *P* < 0.0001; Supplementary Table S6). Short days prior manipulation (SD) had an average total of 126.57 ± 3.61 active min/day. Testosterone treatment during short-day photoperiod (SD + T) had an average of 129.33 ± 2.57 active min/day. When moved to long day photoperiods (LD), average total active minutes per day increased to 159.57 ± 2.96.

### Relationship between basal metabolic rate and mitochondrial DNA copy number

The model investigating relationships between mass corrected BMR and RBC mtDNAcn also found a significant effect of treatment on BMR (F_2, 28.74_ = 8.34, *P* = 0.0014: Supplementary Fig. S2A) and also found a significant interaction between treatment and RBC mtDNAcn (F_2, 28.58_ = 4.77, *P* = 0.0164). No main effect of RBC mtDNAcn on mass corrected BMR was observed (F_1, 26.57_ = 0.13 *P* = 0.7235). Pair-wise post-hoc comparisons of the slopes for each treatment period indicated a significant difference between sparrows housed on short days (SD) and after being moved to long days (LD) for mass corrected BMR (Post Hoc: SD-LD t(_df = 29.13_) = 2.96, *P* = 0.0164; Supplementary Table S7). No significant differences in the slopes were found between sparrows housed on short days (SD) and short days with implants (SD + T), along with short days with implants (SD + T) and when birds were moved to long days (LD). Additionally, a significant effect of treatment on non-mass corrected BMR was found. (F_2, 28.03_ = 8.07, *P* = 0.0017: Supplementary Fig. 2B), along with a significant interaction between treatment and RBC mtDNAcn (F_2, 27.99_ = 4.47, *P* = 0.0206). No main effect of RBC mtDNAcn on non-mass corrected BMR was observed (F_1, 23.36_ = 0.006 *P* = 0.9414). Pair-wise post-hoc comparisons of the slopes for each treatment period indicated a significant difference between sparrows housed on short days (SD) and after being moved to long days (LD) for non-mass corrected BMR (Post Hoc: SD-LD t(_df= 28.59_) = 2.86, *P* = 0.0206; Supplementary Table S8). No significant difference in the slopes were found between sparrows housed on short days (SD) and shorts day with implants (SD + T), along with shorts days with implants (SD + T) and when birds were moved to long days (LD) for non-mass corrected BMR.

### Repeatability of basal metabolic rate and mitochondrial DNA copy number

**Fig. 5:**
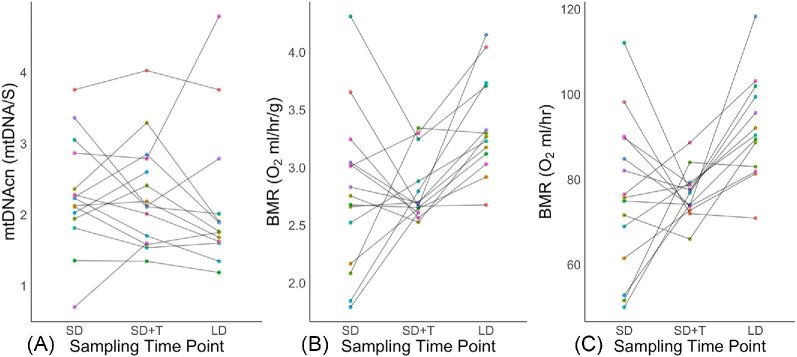
(A) Levels of RBC mtDNAcn across the three treatment groups per individual (Short days [SD]; Short days with testosterone implants (SD + T); Long days [LD]). Individuals are denoted by specific colors that are consistent across each sample period indicating when RBC mtDNAcn was taken. (B) Individual mean BMR corrected with mass across the three treatment groups per individual (Short days [SD]; Short days with testosterone implants (SD + T); Long days [LD]). Individuals are denoted by specific colors that are consistent across each sample period indicating when BMR was measured. (C) Individual mean non-mass corrected BMR across the three treatment groups per individual (Short days [SD]; Short days with testosterone implants (SD + T); Long days [LD]). Individuals are denoted by specific colors that are consistent across each sample period indicating when BMR was measured. Data points represent the average of the two lowest BMR measurements from the last half the BMR measuring time frame for each individual for each treatment period.

Mitocondrial DNA copy number of red blood cells (RBC mtDNAcn) was highly repeatable across all three treaments: (R = 0.631, 95% CI: 0.269–0.803; Fig. 5A; Table 1). However, both mass corrected (R = 0.112, 95% CI: 0.0–0.365; Fig. 5B; Table 1) and non-mass corrected (R = 0.051, 95% CI: 0.0–0.309; Fig. 5C; Table 1) BMR was not repeatable across all three treatments. Additionally both mass corrected (R = 0.11, 95% CI: 0.0–0.545; Table 1) and non-mass corrected (R = 0.0, 95% CI: 0.0–0.486; Table 1) BMR was not repeatable between short days and short days with implants, as well as not being repeatable between short days with implants and long days (Mass corrected; R = 0.244, 95% CI: 0.0–0.538; Table 1; Non-mass corrected; R = 0.142, 95% CI: 0.0–0.410; Table 1).

**Table 1:** Repeatability (R) for RBC mtDNAcn (mtDNA/S), mass corrected BMR (O_2_ mL/h/g), and non-mass corrected BMR (O_2_ mL/h), with the variances for between (V_I_) and within individual (V_W_), as well as the variances of fixed effects in the models (V_F_) across all treatments (short days, short days with testosterone implants and long days) with 95% confident intervals (CI). nBoot is the number of bootstraps performed for each model. Additionally, repeatability was measured for mass corrected BMR (O_2_ ml/hr/g), and non-massed corrected BMR (O_2_ ml/hr) between short days and short days with testosterone implants, as well as between short days with testosterone implants and long days.

Variable	nBoot	R	V_I_	V_F_	V_W_	95% CI
RBC mtDNAcn	1000	0.631	0.477	0.035	0.244	(0.269, 0.803)
BMR (O_2_ mL/h/g)	1000	0.112	0.035	0.069	0.206	(0.0, 0.365)
BMR (O_2_ mL/h)	1000	0.051	11.292	53.513	155.942	(0.0, 0.309)
Short days-short days T						
BMR (O_2_ mL/h/g)	1000	0.11	0.029	0.0	0.234	(0.0, 0.545)
BMR (O_2_ mL/h)	1000	0.0	0.0	0.065	177.109	(0.0, 0.486)
Short days T-long days						
BMR (O_2_ mL/h/g)	1000	0.244	0.05	0.079	0.076	(0.0, 0.538)
BMR (O_2_ mL/h)	1000	0.142	20.952	63.532	63.495	(0.0, 0.410)

## Discussion

Our study asked if testosterone, independent of current reproductive state and under thermoneutral conditions, increased BMR, and if this change in BMR was driven by an increase in red blood cell mitochondrial density (RBC mtDNAcn). We found that experimentally elevated levels of circulating testosterone under non-breeding short photoperiods did not increase BMR. However, following photostimulation on long days, BMR was significantly increased. Additionally, neither endogenous (photostimulated) nor exogenous (implants) sources of testosterone affected blood RBC mtDNAcn, although RBC mtDNAcn had high repeatability across sampling time points. Interestingly, compared to previous studies performed in several avian species (Bech et al. 1999; Rønning et al. 2005; Versteegh et al. 2008; Broggi et al. 2009), BMR in our study had a low repeatability across treatment sampling points. However, these studies investigated the repeatability of BMR over one seasonal time point or at constant lighting regimes. In fact, repeatability of BMR is lower under field conditions potentially due to changes in environmental factors (Auer et al. 2016), and the changing photoperiods in our study may explain why we observed low repeatability of BMR.

Contrary to our hypothesis that testosterone would alter BMR regardless of reproductive state (i.e., regresssed vs. enlarged testes), we observed that BMR was only significantly increased under long-day photoperiods. Photoperiodic increases in BMR have been exhibited in several bird species under longer daylengths, which often corresponds with the breeding season and elevation of endogenous sex hormones, as well as being reproductively receptive (Weathers & Sullivan 1993; Nilsson & Råberg 2001; Anava et al. 2002; Smit & McKechnie 2010; Swanson 2010). Testosterone implants under non-breeding short-day photoperiods in our study did not increase BMR, indicating that testosterone is not directly affecting BMR, at least in birds held on short-day photoperiods. Similar results were exhibited in hormonally manipulated non-breeding male house sparrows in Buttemer et al. (2008), where experimentally elevated levels of testosterone had no treatment effect on BMR. However, birds in Buttemer et al. (2008) were only kept under short-day non-breeding conditions and BMR under long-day conditions were not investigated in their study.

The lack of effect of testosterone implants on BMR during short non-breeding photoperiods in our study could be a result of a seasonal reduction in androgen receptor density across tissues decreasing sensitivity to testosterone (Prins et al. 1990; Stankov et al. 1992; Thaka et al. 1997; Bilinska et al. 2001; Tetel et al. 2004; Leska et al. 2012). In fact, several tissue types (i.e., liver, intestines, and muscle) exhibit a seasonal metabolic reorganization and changes in tissue metabolic capacities, along with seasonal variation in organ mass (Kiss et al. 2009; Swanson 2010; Thibault et al. 1997), potentially contributing to seasonal fluctuations in BMR. These tissue specific changes in metabolic activity could potentially be driven by changes in tissue sensitivity to reproductive hormones. For example, in the avian brain, photoperiod has been indicated to influence tissue specific androgen sensitivity, via changes in androgen receptor densities, allowing for neuromodulation and neurogenesis to alter physiological responsivity and behaviors, such as song, at different times of the year (Bernard & Ball 1997; Brenowitz & Larson, 2015; Fraley et al. 2010; Smith et al. 1995, Smith et al. 1997; Soma et al. 1999).

Conversely, in our study CP size robustly responded to testosterone implants during short daylengths, indicating this tissue retains androgen sensitivity throughout the year. Paired testes size during this time frame, however would have remained relatively small and non-functional due to the lack of photoperiod stimulation and downregulation of androgen receptors under short photoperiods (Thaka et al. 1997; Bilinska et al. 2001; Leska et al. 2012). Additionally, negative feedback effects on the hypothalamus and pituitary gland by increased levels of androgens could lead to the suppression of gonadal growth as seen in both intact male Japanese quail (Brown & Follett 1977) and in breeding male white-plumed honeyeaters (*Lichenostomus penicillatus*)(Buttemer & Astheimer 2000) treated with testosterone manipulations. On the contrary it is important to note that testes volume did increase marginally when male rufous collared sparrows (*Zonotrichia capensis*) (Small et al. 2015) and house finches (*Carpodacus mexicanus*) (Hamner 1968; Deviche et al. 2006) received testosterone implants on reproductively inhibiting daylengths. While increases in testes size were exhibited, they remained small in comparison to breeding testes size (Hamner 1968; Stevenson et al. 2012). It is not known how this relatively small increase in testis size (compared with size during breeding season) contributes metabolically to whole organismal BMR. We did not measure testis size until the end of the experiment, and cannot be certain of the state of testes following testosterone treatment nor its contribution to the lack of effect of testosterone treatment while on short-days on BMR in the current study. As indicated above, metabolic changes in BMR may be due to changes in tissue (i.e., liver, muscle, brain, and intestinal) specific androgen sensitivity via photoperiodic regulation of androgen receptors. However, it remains under study which somatic tissues, other than reproductive tissues, experience changes in photoperiodic androgen sensitivity or how much these tissue-specific changes contribute to BMR and metabolic activity. Additionally, to our knowledge, most studies investigating the effects of sex hormones on metabolic activity focus on a single time point within a season or manipulation under a single lighting regime, potentially neglecting changes in hormone sensitivity.

The maintenance and seasonal changes in the presence or absence of mature gonads (metabolically costly tissues) could be a potential mechanism for seasonal changes in metabolic rate. A reduction in BMR post-castration in male Japanese quail (*Coturnix japonica*), male spotted munia (*Lonchura punctulate*) and male red-headed buntings (*Emberiza bruniceps*) exposed to long-day photoperiods has been reported (Hanssler I & Prinzinger R 1979; Thapliyal et al. 1983; Gupta & Thapliyal 1984). These studies further indicate that BMR was not restored to pre-castration levels with subsequent testosterone replacement (Hanssler & Prinzinger 1979; Thapliyal et al. 1983; Gupta & Thapliyal 1984). These observations, combined with our study in which birds after photostimulation had paired testes size that was comparable to paired testes size in breeding wild house sparrows (Lombardo & Thorpe 2009), suggest that gonadal maturation and maintenance of functional gonads, and not testosterone per se, may be one factor playing a role in seasonal changes of BMR. In fact, in previous studies gonadal mass and size were positively related to BMR during the breeding season and found to be a predictor of BMR and daily energy expenditure across several species (Meerlo et al. 1997; Chappell et al. 1999; Vézina & Williams 2003; Kaseloo et al. 2012; Padilla et al. 2023). Furthermore, Chappell et al. (1999) indicated that male house sparrows experience a 19–29% increase in BMR when testes size increased during the breeding season compared to the non-breeding season (lack of matured testes). In our study, we do see that BMR was only increased under long days when matured testes were present, adding evidence complementing these studies that gonadal growth could be driving seasonal changes in metabolic rate. Additionally, our study tried to uncouple the presence/absence of functional gonads and the effects of testosterone by sampling birds held under short days with testosterone manipulation and also sampled under long days with testosterone produced by functional reproductive testes. During short days with implants, as noted above, paired testes size would have likely remained non-functional due to the lack of photoperiodic stimulation or increased negative feedback effects on the reproductive axis caused by experimentally elevated levels of testosterone. While under long days testes volume would have increased and been fully functional due to photostimulation leading to an increase in endogenous androgens. Having these time points allowed us to uncouple the effects of testosterone and the presence of functional gonadal tissue on BMR and indicate that having matured testes and not testosterone, may be a contributing factor in seasonal changes in BMR. However, to fully tease apart the relationship between reproductive hormones and gonadal maturation on BMR future studies should include intact and castrate groups.

In addition to increased energy directed towards maintaining functional testes, the longer daylight hours may also have contributed to the long-day induced increase in BMR via increased duration of daily activity. In our study, total daily activity was not altered in response to testosterone treatment under short-days compared with duration of activity prior to implantation, contrary to our predictions that activty would be increased with implants due to advanced waking times during short days following testosterone treatment (Galante et al. 2024). However, we observed that both duration (how long they were active) and amount of total daily activity did increase following transfer to long days. Galante et al. (2024), using the same birds as in this current study, indicated that the total activity % relative to day length, a relative measure of activity intensity, decreased when birds were transferred to long days, strengthing the relationship between increased total activity due to longer photoperiods and increased BMR. The postive relationship between increased activity and BMR have been in exhibted across several species as well. For instance, in male white-footed mice, locomotor activity during metabolic measurement, was positively correlated with both metabolic rate above rest (average daily metabolic rate-BMR) (Kaseloo et al. 2012) and average daily metabolic rate, regardless of hormone manipulation (McDonnell et al. 2019). Additionally, our observations match Wikelski et al. (1999), where both castrated and intact, white-crowned sparrow (*Zonotrichia leucophrys*) males that were transitioned from short photoperiods to long photoperiods displayed an increase in resting metabolic rate and total daily activity (hops/day) prior to hormone manipulation. These observations combined with our data indicate that photoperiod-related increased activity is positively associated with increased metabolic rate, regardless of reproductive state. However, we do note that unlike the study by Wikelski et al. (1999), we were unable to include castrated birds with and without testosterone implants under long days to fully disentangle the effects of long-day increases in activity, developed testes, and testosterone on BMR. Alternatively, elevated BMR relative to photoperiod induced increases in total activity could be reflecting the changes in body composotions of central organs along with changes in organ specfic metabolic rates (i.e., increased in skeletal/pectoralis muscle, gut, liver, kindey, and heart) to account and maintain the cost for overall increases in engery demand (Chappell et al. 1999; Swanson 2010).

Previous studies that have focused on the impact of long-term daily exercise on BMR in animal models, indicate that increased activity increases BMR potentially to offset the cost of the oxygen debt experienced from the thermic effect of exercise (Gleeson et al. 1982; Hill et al. 1984; Wilterdink et al. 1993; Pinto & Shetty 1995; Ichikawa et al. 2000; Speakman & Selman 2003). For example, in free-living northern gannets (*Sula bassanus*) flight duration (h/day) was positively correlated with field metabolic rate (Birt-Friesen et al. 1989). Additionally, a 10% increase in resting metabolic rate was exhibited in rats that ran daily for 1 h at a speed of 0.9 km/h on a treadmill for 56 days (Gleeson et al. 1982). In humans constant elevated exercise, cycling or jogging/walking 4–6 times weekly for 30–40 min sessions (Menshikova et al. 2006) and swimming between 36 and 15 km weekly (Baykara et al. 2016) upregulated mtDNAcn found in skeletal muscle and peripheral blood lymphocytes, potentially due to increased mitochondrial biogenesis (Menshikova et al. 2006; Baykara et al. 2016). Additionally, oxidative phosphorylation enzymes, cytochrome-c oxidase and citrate synthase, in skeletal muscle, were increased in relation to exercised (i.e., wheel or treadmill running) versus non exercised groups in rodents (Moraska et al. 2000; Houle-leroy et al. 2003). These results, combined with our lack of change in RBC mtDNAcn, indicate that cellular mechanistic changes that the mitochondria experiences in relation to exercise, likely in tissues/organs altered by the exercise (and not in other tissues like RBCs), provides potential pathways for how exercise can impact BMR and metabolic activity.

Opposite our predictions, we did not observe increases in RBC mtDNAcn with endogenous (photostimulation) or exogenous treatment-induced increases in testosterone. Interestingly, we observed that individual RBC mtDNAcn was highly repeatable while BMR was not. The low repeatability of BMR could be a result of the effect of photoperiod on gonadal tissue growth along with individual differences in responsiveness of their reproductive axis. Moreover, differences in individual activity across treatments and difference in food intakes, may have contributed as well to the low repeatability of BMR in our study. The high repeatability of RBC mtDNAcn indicates that within individual variation in RBC mtDNAcn levels was lower than variation between individuals across treatments (i.e., a bird with higher RBC mtDNAcn remained high relative to other individuals across treatments). Blood mtDNAcn was also repeatable in the pied flycatcher under natural breeding conditions (Stier et al. 2019), and the repeatability we observed is within the range reported by a meta-analysis on mitochondrial traits (Nespolo & Franco 2007), indicating RBC mtDNAcn as a stable trait. While the repeatability of RBC mtDNAcn is high, much remains unknown about how genetic vs. environmental effects impact mtDNAcn in the blood along with other tissues (Dillin et al. 2002; Toews et al. 2014; Stier et al. 2019).

Contrary to our prediction that elevation in testosterone would increase RBC mtDNAcn and this increase would be related to an increase in BMR, we did not observe treatment effects on RBC mtDNAcn. Thus, the treatment effects in BMR seen in our study are not explained by RBC mtDNAcn, indicating that RBC mtDNAcn is not a good metabolic indicator for whole organismal BMR. However, it remains under investigation if mtDNAcn from other metabolically active tissues (i.e., skeletal muscles, liver, and gonads) are potentially better metabolic indicators, since it has been indicated that tissue specific metabolic activities contribute to whole organismal metabolic rates disproportionally (Meerlo et al. 1997; Vézina & Williams 2003; Padilla et al. 2023). However, to achieve assessment of non-blood mtDNAcn often requires terminal sampling. To enable repeated non-lethal sampling in our study, mtDNAcn was obtained from nucleated red blood cells. Our lack of treatment effects on mtDNA could be a result of blood mtDNAcn not representing whole organismal BMR or cellular respiration but rather reflecting current blood composition and humoral factors (Picard 2021). Additionally, blood makes up a small percentage of body composition, and it remains unknown what percentage blood plays in whole organismal metabolic rate. Alternatively, RBC mtDNAcn may be related with other relevant metabolic traits, such as summit metabolic rate, rather than BMR (Swanson 2010).

While we found that RBC mtDNAcn was not related with BMR, other mitochondrial traits have been related with BMR. Blood mitochondrial respiration, which allows for direct measurement of cellular oxidative phosphorylation, is a strong indicator of whole organism BMR and moderately mirrors cell respiration in other tissues (Stier et al. 2017, 2019; Casagrande et al. 2023; Thoral et al. 2024). Thus, mitochondrial respiration could serve as a better unit of measure than RBC mtDNAcn when investigating the pathways that underlie the seasonal variation in BMR. While blood mitochondrial respiration may be a better gauge of both cellular and organismal respiration, there are limitations to this technique that may not be suitable for most field studies (i.e., most samples need to be run within 24 h of being collected) (Stier et al. 2017). Additionally, whole organismal metabolic rates may be hard to acquire and often not accessible when working in field environments. Therefore, there is a need to develop a quick and minimally invasive sampling technique where samples can be stored for long periods of time which measures aspects of organismal metabolic rates. Potentially, mtDNAcn could fill this gap, however, if blood mtDNAcn is to be useful as data, it is critical to understand what blood mtDNAcn is indicating. To achieve this, future studies should investigate the relationship between blood mtDNAcn, along with other tissues, and mitochondrial respiratory capacities, in conjunction with summit, average daily and above rest organismal metabolic rates (Picard 2021).

Finally, while our study examined the effects of testosterone on BMR across different breeding conditions and found no effect, past studies have returned conflicting results regarding testosterone’s influence on BMR and activity, suggesting more work is required. Wikelski et al. (1999) found in both intact and castrated, white-crowned sparrows, a migratory species, that testosterone administration decreased resting metabolic rate while increasing the activity level only under long photoperiods. However, Buttemer & Astheimer (2000) and Buchanan et al. (2001) showed contrasting effects of testosterone treatment on BMR in two species of non-migratory birds, house sparrows and white-plume honey eaters. Testosterone treatment did not affect BMR in breeding male white-plumed honeyeaters (Buttemer & Astheimer 2000) and testosterone treatment during autumn molt increased BMR in male house sparrows (Buchanan et al. 2001). These results indicate that testosterone is having vastly different physiological functions between species, life history stages, and metabolic activity type. To potentially remedy these differential effects of testosterone on metabolic rate, more studies are needed that investigate impacts of testosterone on BMR across several different species (i.e., non-migratory vs. migratory), at different time points within and across different life history stages, and across different metabolic activity measurements (i.e., submit, resting, and BMR).

## Conclusion

While birds were under non-breeding short days, elevated levels of testosterone did not increase BMR; however, photostimulation did elevate BMR. These findings suggest that testosterone is not directly responsible for increasing BMR during seasonal reproduction. Additionally, we observed that total daily activity was only increased under longer photoperiods, indicating that there was no treatment effect of testosterone implants on activity. Combined, our data suggest that elevated BMR observed during the reproductive period may better reflect energetic investments in reproductive tissues and/or the longer daylight hours may also have contributed to the long day induced increases in BMR via increased daily activity. Unexpectedly, there was no treatment effect on blood mtDNAcn; however, blood mtDNAcn was highly repeatable across treatments per individual, mirroring similar results found in the pied flycatcher where blood mtDNAcn was repeatable across breeding stages. Our result indicates that changes in BMR are not acting through mtDNAcn, or at least not blood mtDNAcn. Future research is needed to understand what blood mtDNAcn is indicating and its contribution to both cellular and organismal respiration (i.e., summit, average daily and above rest metabolic rates) to develop more field-friendly techniques to measure metabolic capacities. Overall, our study indicates that testosterone is not indirectly affecting BMR through changes in mitochondrial density, however changes in photoperiod are directly affecting BMR potentially by increasing daily activity or by stimulating the growth of energetically costly gonadal tissue in long day seasonally breeding birds.

## Supplementary Material

obag013_Supplemental_File

## Data Availability

The data that support the findings of this study are available from the corresponding author upon reasonable request.
