# Supplementary material for: Photoperiod, but not testosterone, increases basal metabolic rate in house sparrows
Source: Integr Org Biol. 2026 Apr 2;8(1):obag013. doi: 10.1093/iob/obag013 (PMC13083693; doi:10.1093/iob/obag013)
Supplement: obag013_Supplemental_File [file obag013_supplemental_file.docx]

Supplemental Table 1: Mean and standard error of the mean (SEM) for blood plasma levels of testosterone (ng mL^-1^ ) for each treatment (Galante et al., 2024).

| **Treatment** | **Mean (**ng mL^-1^) | **SEM** |
| --- | --- | --- |
| SD | 0.11 | 0.01 |
| SD+T | 13.22 | 1.84 |
| Post T SD | 0.36 | 0.06 |
| LD | 2.22 | 0.47 |

Supplemental Table 2: Primer sequences for mitochondrial gene Cytochrome B (CytoB) and nuclear gene Glyceraldehyde-3-phosphate dehydrogenase (GAPDH). Sequences for GAPDH were previously validated by Heidinger et al. (2021) and Velando et al. (2019). Sequences for CytoB were designed from the complete annotated house sparrow mitochondrion found on NCBI (KM078784.1).

| GAPDH Forward | 5′-AACCAGCCAAGTACGATGACAT-3′ |
| --- | --- |
| GAPDH Reverse | 5′-CCATCAGCAGCAGCCTTCA-3′ |
|  |  |
| CytoB Forward | 5’-GCCCTCTATCCCAAATCCTATTC-3’ |
| CytoB Reverse | 5’-AAGTAGGAGAGTGAGGCTAGTT-3’ |

Supplemental Table 3: Pair-wise post-hoc comparisons of CP size (mm) across each treatment using a Holm-Bonferroni adjustment.

| **Contrast** | **Estimate** | **SE** | **df** | **t ratio** | ***p value*** |
| --- | --- | --- | --- | --- | --- |
| SD - (SD+T) | -1.49 | 0.10 | 39.08 | -15.02 | <0.0001 |
| SD - Post T SD | -0.13 | 0.10 | 39.64 | -1.29 | 0.2047 |
| SD - LD | -1.27 | 0.10 | 40.02 | -12.23 | <0.0001 |
| (SD+T) - Post T SD | 1.36 | 0.10 | 39.64 | 13.40 | <0.0001 |
| (SD+T) - LD | 0.22 | 0.10 | 40.02 | 2.11 | 0.0814 |
| Post T SD - LD | -1.14 | 0.10 | 39.48 | -10.85 | <0.0001 |

Supplemental Table 4: Pair-wise post-hoc comparisons of massed corrected BMR (O_2_ ml/hr/g) across each treatment using a Holm-Bonferroni adjustment.

| **Contrast** | **Estimate** | **SE** | **df** | **t ratio** | ***p value*** |
| --- | --- | --- | --- | --- | --- |
| SD - (SD+T) | -0.02 | 0.17 | 26.11 | -0.11 | 0.9098 |
| SD - LD | -0.57 | 0.17 | 27.19 | -3.31 | 0.0079 |
| (SD+T) - LD | -0.56 | 0.17 | 27.19 | -3.20 | 0.0079 |

Supplemental Table 5: Pair-wise post-hoc comparisons of non-mass corrected BMR (O_2_ ml/hr) across each treatment using a Holm-Bonferroni adjustment.

| **Contrast** | **Estimate** | **SE** | **df** | **t ratio** | ***p value*** |
| --- | --- | --- | --- | --- | --- |
| SD - (SD+T) | -0.39 | 4.57 | 26.01 | -0.08 | 0.9334 |
| SD - LD | -15.93 | 4.76 | 27.02 | -3.35 | 0.0072 |
| (SD+T) - LD | -15.55 | 4.77 | 26.84 | -3.26 | 0.0072 |

Supplemental Table 6: Pair-wise post-comparisons of total active minutes per day across each treatment using a Holm-Bonferroni adjustment.

| **Contrast** | **Estimate** | **SE** | **df** | **t ratio** | ***p value*** |
| --- | --- | --- | --- | --- | --- |
| SD - (SD+T) | -4.32 | 3.19 | 578.45 | -1.36 | 0.1759 |
| SD - LD | -38.67 | 3.54 | 583.32 | -10.91 | <0.0001 |
| (SD+T) - LD | -34.35 | 3.33 | 583.71 | -10.32 | <0.0001 |

Supplemental Table 7: Pair-wise post-comparisons of the correlational slopes between RBC mtDNAcn and massed corrected BMR (O_2_ ml/hr/g) for each treatment period using the “pairs” function from “emtrends”.

| **Contrast** | **Estimate** | **SE** | **df** | **t ratio** | ***p value*** |
| --- | --- | --- | --- | --- | --- |
| SD - (SD+T) | 0.47 | 0.22 | 27.54 | 2.16 | 0.0954 |
| SD - LD | 0.57 | 0.19 | 29.13 | 2.96 | 0.0164 |
| (SD+T) - LD | 0.11 | 0.20 | 28.37 | 0.54 | 0.8532 |

Supplemental Table 8: Pair-wise post-comparisons of the correlational slopes between RBC mtDNAcn and non-massed corrected BMR (O_2_ ml/hr) for each treatment period using the “pairs” function from “emtrends”.

| **Contrast** | **Estimate** | **SE** | **df** | **t ratio** | ***p value*** |
| --- | --- | --- | --- | --- | --- |
| SD - (SD+T) | 12.32 | 5.86 | 27.57 | 2.10 | 0.1079 |
| SD - LD | 15.03 | 5.25 | 28.59 | 2.86 | 0.0206 |
| (SD+T) - LD | 2.71 | 5.34 | 27.99 | 0.51 | 0.8679 |


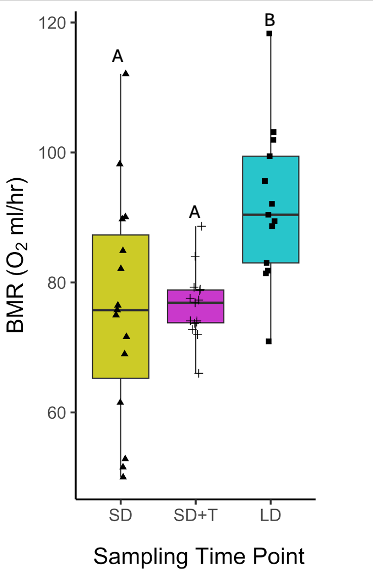


Supplemental Figure 1: Individual mean of non-massed corrected BMR across treatments (Short days (SD); Short days with testosterone implants (SD+T); Long days (LD)). Data points represent the average of the two lowest BMR measurements from the last half of the BMR measuring time frame for each individual for each treatment period. Letters indicate significant differences between groups


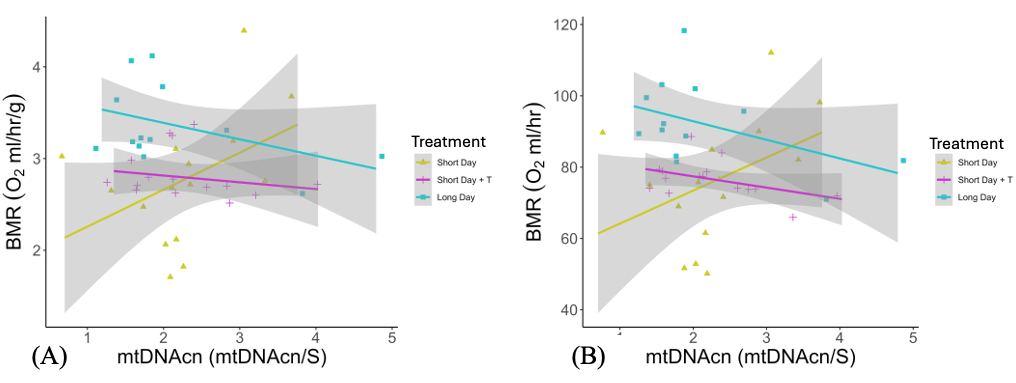


Supplemental Figure 2: The correlation between mass corrected (A) and non-mass corrected (B) BMR and RBC mtDNAcn for each treatment period Short days (SD); Short days with testosterone implants (SD+T); Long days (LD)). Data points represent a single individual for each sampling time point for RBC mtDNAcn, mass corrected and non-mass corrected BMR. Both mass corrected and non-mass corrected BMR was calculated using the two lowest BMR measurements from the last half the BMR measuring time frame for each individual for each treatment period.
